# No Apparent Influence of Reward upon Visual Statistical Learning

**DOI:** 10.3389/fpsyg.2016.01687

**Published:** 2016-11-02

**Authors:** Leeland L. Rogers, Kyle G. Friedman, Timothy J. Vickery

**Affiliations:** Department of Psychological and Brain Sciences, University of Delaware, NewarkDE, USA

**Keywords:** statistical learning, reward processing, reward learning, visual attention, associative learning, implicit learning

## Abstract

Humans are capable of detecting and exploiting a variety of environmental regularities, including stimulus-stimulus contingencies (e.g., visual statistical learning) and stimulus-reward contingencies. However, the relationship between these two types of learning is poorly understood. In two experiments, we sought evidence that the occurrence of rewarding events enhances or impairs visual statistical learning. Across all of our attempts to find such evidence, we employed a training stage during which we grouped shapes into triplets and presented triplets one shape at a time in an undifferentiated stream. Participants subsequently performed a surprise recognition task in which they were tested on their knowledge of the underlying structure of the triplets. Unbeknownst to participants, triplets were also assigned no-, low-, or high-reward status. In Experiments 1A and 1B, participants viewed shape streams while low and high rewards were “randomly” given, presented as low- and high-pitched tones played through headphones. Rewards were always given on the third shape of a triplet (Experiment 1A) or the first shape of a triplet (Experiment 1B), and high- and low-reward sounds were always consistently paired with the same triplets. Experiment 2 was similar to Experiment 1, except that participants were required to learn value associations of a subset of shapes before viewing the shape stream. Across all experiments, we observed significant visual statistical learning effects, but the strength of learning did not differ amongst no-, low-, or high-reward conditions for any of the experiments. Thus, our experiments failed to find any influence of rewards on statistical learning, implying that visual statistical learning may be unaffected by the occurrence of reward. The system that detects basic stimulus-stimulus regularities may operate independently of the system that detects reward contingencies.

## Introduction

At every moment, human cognition faces the complex task of interpreting and responding to an overwhelming amount of stimulation. One important means by which humans may cope with this constant stream of information in the world is by learning and exploiting statistical regularities ubiquitous in natural environments. Many laboratory studies have demonstrated the potential for human learning to pick up such regularities in an unsupervised fashion. For example, repeatedly experiencing one phoneme that reliably predicts another (Saffran et al., 1996), or particular visual items that reliably co-occur in time or space with others (Fiser and Aslin, 2001, 2002), can lead to above-chance recognition rates of those regularities. This kind of statistical learning is available to us from a time shortly after birth and throughout adulthood (Saffran et al., 1996; Saffran et al., 1999), and such learning spans perceptual systems (Glicksohn and Cohen, 2013), allowing humans to automatically detect and learn rich probabilistic relationships common within real-world environments. Although statistical learning can be more complex, simple stimulus-stimulus associative relationships are an important (and most commonly studied) component of statistical learning, and these relationships can apparently be detected and learned without observers’ intentions or awareness (Turk-Browne et al., 2005).

There is no shortage of evidence that visual statistical learning is a powerful and ubiquitous ability in humans (Turk-Browne and Scholl, 2009; Turk-Browne et al., 2009; Glicksohn and Cohen, 2011) as measured by recognition or familiarity with contingent stimuli. However, the consequences of experiencing statistical regularities in our environment is not limited to simply making sense of streams of information by segmenting and chunking stimuli. For instance, performance is improved for target items that are predicted by preceding elements in a visual statistical learning stream (Turk-Browne et al., 2010). Evidence also suggests that statistical regularities bias attention, with attention being drawn to regions in which statistical regularities occur (Zhao et al., 2013). Attention can also be guided to locations based upon implicitly learned associations between distractor and target positions (Chun and Jiang, 1998). Thus, visual statistical learning is considered useful in new environments for both recognition and for the guidance of attention.

Another type of associative learning that also possesses the capacity to guide attention is stimulus-reward learning. Reliable associations between stimuli and rewards have been shown to influence performance in many different contexts, and there is a rich history of animal studies showing strong influences of primary reward associations (Schultz et al., 1997; Berridge, 2007; Haber and Knutson, 2009) guiding behavior and driving brain activity, where primary rewards are water, juice, or food rewards that are directly registered as rewards by brain circuits as a function of states such as thirst and hunger. However, secondary rewards (e.g., money, or stimuli indicating monetary value or simply “positive” outcomes) can also be highly effective at driving performance and brain activity, and are effective as stimuli which, when reliably paired with a previously non-rewarding stimulus, imbue that previously non-rewarding stimulus with value (Daw and Doya, 2006; Haber and Knutson, 2009).

A large and growing literature using human subjects has employed such secondary cues in order to imbue previously non-rewarding stimuli with value, leading to striking differences in performance related to differences in stimulus-value associations. Higher (explicitly learned) associative value, based on secondary reward in terms of monetary value, leads to better explicit recognition memory, and high value associations can even lead to stimuli escaping the attentional blink (Raymond and O’Brien, 2009), suggesting that such associations drive low-level attentional biases. Even in cases where participants are not consciously aware of the association between stimulus characteristics and rewarding outcomes, evidence suggests a clear attentional bias toward stimuli that are consistently paired with higher secondary rewards (Anderson et al., 2011; Sha and Jiang, 2015). Stimulus-reward learning may allow for the optimization of behavior by automatically orienting attention towards reward-predicting elements of a scene, and thus help optimize choice behavior to seek reward and avoid punishment (Engelmann et al., 2009; Hickey et al., 2010; Theeuwes and Belopolsky, 2012; Chelazzi et al., 2013; Sali et al., 2014; Pessoa, 2015). Mounting evidence suggests that even secondary cues to value can serve as associative markers that drive value-based differences in low-level performance.

These two types of learning, statistical learning and stimulus-reward learning based upon secondary reward cues, bear some obvious similarities. Visual statistical and reward learning mechanisms incorporate similar associative mechanisms. Indeed, in many published cases in which stimulus-reward learning plays a significant role, a visual stimulus is typically repeatedly paired with another specific sensory stimulus that indicates reward value – thus, both statistical learning and reward-related learning could play a role in such studies. As reviewed above, both types of learning appear to play a role in biasing selective attention. Studies of the neural bases of these mechanisms provide further reason to suspect that they may be interrelated. Visual statistical learning may be supported by some of the same neural structures that support reward learning; correlates of both reward learning and visual statistical learning have been noted in striatum and medial temporal lobe structures (Delgado et al., 2000; Aron, 2004; Wittmann et al., 2007), and there is increasing evidence that the hippocampus plays a role in reward learning as well as trial-and-error learning (Lansink et al., 2009; Dickerson and Delgado, 2015). Thus, the visual statistical learning system bears some resemblance to the value-learning system, in terms of the importance of prediction and deviations from predicted events in generating surprise signals, but the relationship between the systems is currently not well characterized.

Given the similar nature of these two types of learning and how they appear to contribute to our ability to learn about and navigate environments, an intuitive question arises - how do these two mechanisms interact? To our knowledge, potential relationships between reward and visual statistical learning remain unexplored. Even if they do not depend upon shared mechanisms, there is reason to believe that they may interact. To wit, evidence suggests that statistical learning is dependent upon selective attention to the constituent, related items (Turk-Browne et al., 2005). If rewarding events drive attention (Jiang et al., 2013), then stimulus-stimulus learning might reasonably be expected to show a dependence upon co-occurrence of constituent stimuli with rewarding events, with more-rewarding events drawing greater attention and leading to stronger memory traces. On the other hand, rewarding events and contingencies might draw attention away from stimulus-stimulus relationships, or occupy resources otherwise required for stimulus-stimulus associative learning, thus impairing such learning.

The current set of experiments seeks to identify whether statistical learning operates independently or not from reward. Specifically, by introducing rewarding events and stimulus-reward associations while simultaneously establishing stimulus-stimulus statistical associations (Experiment 1), or by establishing reward associations immediately before establishing statistical associations (Experiment 2), we sought evidence that reward associations either enhance or impair the ability to detect statistical regularities across time.

## General Materials and Methods

### Ethics Statement

These studies were carried out with full review and approval by the Institutional Review Board at the University of Delaware with written informed consent from all participants.

### Participants

A total of 136 University of Delaware students took part in the study in partial fulfillment of course credit. Experiment 1A included 32 participants and Experiment 1B included 43 participants. Experiment 3 included a total of 61 participants divided into three groups: a first position, second position, and third position reward-associate groups. The first position group contained 22 participants, the second position group contained 18 participants, and the third position group contained 21 participants.

### Stimulus Materials

**FIGURE 1:**
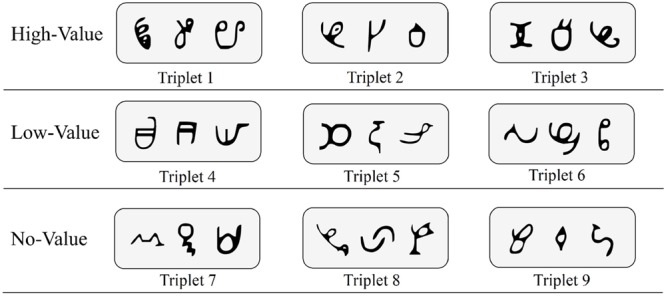
**Sample base triplets of Ndjuká symbols with random assignment to high-value, low-value, and no-value association conditions**.

Visual stimuli were 27 symbols that were novel and unfamiliar to our sample. These symbols, derived from the African Ndjuká syllabary and unfamiliar to our Western subjects, were adopted based upon recent research that successfully utilized them to explore visual statistical learning (Turk-Browne et al., 2009; Zhao et al., 2013; Yu and Zhao, 2015). For every participant, all 27 symbols were randomly assigned to 9 different triplet sets (see **Figure 1** for an example). Triplet sets were then randomly assigned to high-value, low-value, and no-value association conditions (i.e., three triplet sets were assigned to each condition).

### Apparatus

All experiments were run using a computer running Ubuntu Linux and attached to a 17-inch CRT monitor. Experiment 1 was written in Python, using the PsychoPy package (Peirce, 2007) while Experiment 2 was written in MATLAB using the Psychophysics Toolbox extensions (Brainard, 1997; Kleiner et al., 2007).

### Procedure

Participants were given written and oral instructions before each experiment. Critically, participants were only given explicit instructions related to the familiarization phase of the experiment prior to beginning. We intentionally avoided providing any information about the underlying structure (i.e., triplets) within the familiarization phase. Participants were not informed that there would be a test phase following the familiarization phase. After an explanation of the familiarization phase cover task, participants were seated in front of a computer within an isolated and dimly lit testing space. All stages of the experiment were accompanied by a full set of instructions for the participant to read on-screen. Participants were also told to ask the experimenter for clarification on any set of instructions while completing the experiment, as needed.

#### Familiarization Phase

**FIGURE 2:**
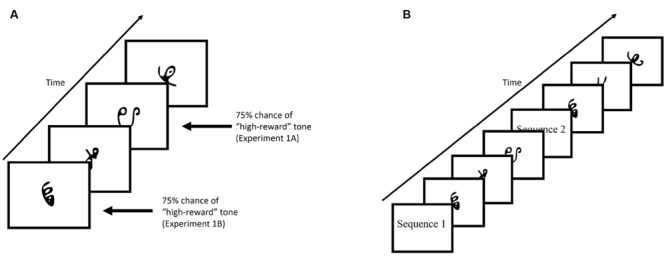
**(A)** Example of the familiarization phase of Experiment 1. Stimuli were presented for 800 ms with a 200 ms blank ITI. **(B)** Example of the test phase. Participants were shown, sequentially, a characters comprised of a target triplet and characters comprised of a foil triplet. Each triplet was preceded by either “Sequence 1” or “Sequence 2”. Participants were then required to select the triplet they viewed during the familiarization phase by pressing “1” or “2” on the keyboard.

**Figure 2A** provides an example of the familiarization phase. Participants viewed a stream of symbol triplets on the computer screen. The presentation of the triplets were randomized. Stimuli sequentially appeared at the center of the screen for 800 ms each, with a 200 ms blank screen inter-trial interval. Each triplet appeared 24 times throughout the familiarization phase, for a total of 648 symbol presentations (not counting 24 1-back repetitions that occurred in Experiment 1). Additionally, participants were required to make responses by pressing the spacebar whenever there was a 1-back repetition (Experiment 1) or if a shape quickly moved back and forth (“jiggle” in Experiment 2). These events occurred 24 times for each individual. Despite the fact that the stream was composed of repetitions of structured triplets containing three symbols, there was no explicit indication that this structure was present. Rather, participants viewed a steady stream of characters throughout the experiment and were expected to implicitly learn the statistical regularities hidden within the stream over the course of the experiment.

In Experiment 1, tones indicating reward status (low-value or high-value), were played through headphones, and occurred on 75% of trials in which a low-value or high-value triplet appeared. In Experiment 1A, the tone always co-occurred with the third item of the triplet sequence, while in Experiment 1B, the tone always co-occurred with the first item of the triplet sequence. In Experiment 2, reward status was established through an explicit reward-learning phase that preceded the familiarization phase (described in section “Experiment 2”).

#### Test Phase

**Figure 2B** provides an example of the test phase. After the familiarization phase and before the test phase, participants were informed that the stream of characters they had just viewed contained structured triplets. The instructions continued on to explain the test phase. On each trial of the test phase, participants were shown two sequences (i.e., triplets) and had to choose the sequence that appeared more familiar to them. At the beginning of the trial, participants viewed the words “Sequence 1” on the screen for 1000 ms, followed by a central fixation cross presented for 500 ms. Three stimuli then appeared on screen with identical timing to the familiarization phase. After the first sequence had completed, a second sequence with the preceding label “Sequence 2” appeared on screen. Participants chose the sequence that appeared more familiar to them by pressing “1” or “2” on the keyboard in front of them.

Test phase trials employed one of the nine original triplets and one of nine foil triplets. Foil triplets were constructed using the same symbols exposed during training, recombined into new triplets such that each shape appeared in the same position within both the original and foil triplet (e.g., first, second, or third item in the triplet), but in novel combinations. For example, given the assignment shown in **Figure 1**, a foil triplet could contain the first character from Triplet 1, the second character from Triplet 2, and the third character from Triplet 3. Foil triplets were constructed exclusively from the same “value” triplets (i.e., we did not intermingle low-reward, high-reward, and neutral triplet constituents in foil triplets). Each trial in the test phase included one original triplet paired with one foil triplet. The order by which an original triplet or a foil triplet appeared was randomized, and participants were again required to choose the triplet that they had observed during the familiarization phase. Experiments 1A and 1B each contained 162 test trials (each triplet was matched with each possible foil exactly twice) while Experiment 2 contained 54 test trials (each triplet was paired against each same-value triplet exactly once). In order to determine if visual statistical learning occurred and reward associations had an impact, proportion correct scores were calculated for each triplet value (e.g., low, high, and neutral), which were compared to chance performance.

## Experiment 1A

Participants were instructed that they would be earning points during the familiarization phase, with the total number of points they earned converted into a cash reward at the conclusion of the experiment. While participants were viewing the stream of symbols, they were told to listen for an occasional “beep”. Every time they heard the high-pitched tone, they would gain 10 points toward their total. Alternatively, if they heard a low-pitched tone, they would not gain any points at all. Critically, and unbeknownst to the participants, these tones could only occur simultaneously with the third item in a triplet (i.e., reward was paired with the triplet a participant had just viewed) 75% of the time. Out of our nine original triplets, we associated a high-value reward with three triplets (+10 each time the high-pitch tone plays), a low-value reward with another three triplets (+0 each time the low-pitch tone plays, and no association with the remaining three triplets. With each triplet being presented 24 times and the high-pitched tone played on 75% of high-value triplet occurrences, each participant earned 540 points (3^∗^(0.75^∗^24)^∗^10 = 540). Points were converted into cents at the end of the experiment, and all participants won a total of $5.40.

Participants were also given an attention-check task to ensure they were paying attention during the familiarization phase of Experiments 1A and 1B. Specifically, whenever a symbol was repeated, participants had to press the spacebar. The third item of each triplet was randomly selected to occasionally repeat throughout the familiarization phase. While reward tones may have occurred with the third item in the triplet, it never occurred with this fourth “repeat” item.

Should the high-pitched tone carrying the high-value association enhance visual statistical learning, then we may expect enhanced recognition of those triplets it had been paired with during the test phase. Alternatively, interference between reward learning and visual statistical learning may be evident if participants are more successful at selecting the low-value or value-absent triplets during the test phase. Finally, results may indicate that reward associations have no impact on visual statistical learning. In other words, despite the co-occurrence of these two powerful types of learning, participants may correctly identify triplets from the familiarization phase evenly across reward conditions.

### Results

**FIGURE 3:**
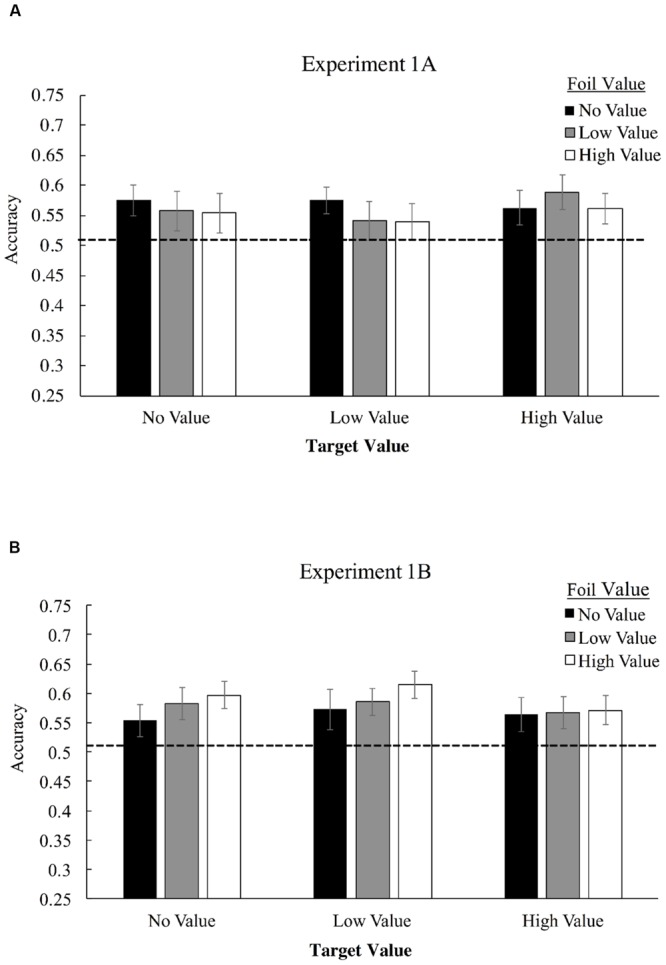
**Mean accuracies in identifying target triplets over foil triplets in Experiment 1A **(A)** and Experiment 1B **(B)**.** High-value triplets (+10 points) consists of shapes which were associated with the high-pitched tone, low value triplets (+0 points) were associated with a low-pitched tone, and value-absent triplets did not possess any association. During Familiarization, only one shape was consistently paired with the tone – the last shape in the triplet for Experiment 1A and the first shape for 1B, but all shapes in the triplet were considered no-, low-, or high-value for purposes of determining foil composition.

**Figure 3A** displays mean accuracies in selecting target triplets over foil triplets for Experiment 1A, as a function of both target and foil triplet value. Employing a 3 × 3 (triplet value × foil value) repeated-measures ANOVA, we found no significant interaction of target triplet value and foil triplet value, *F*(4,124) = 0.65, *p* = 0.63, η_p_^2^ = 0.02, and no main effect of target value association, *F*(2,62) = 0.36, *p* = 0.70, η_p_^2^ = 0.012, or foil value association, *F*(2,62) = 0.38, *p* = 0.69, η_p_^2^ = 0.012. Visual statistical learning, however, was robust as measured by a one-sample *t*-test comparing performance to chance (50% correct), with participants correctly identifying more target triplets overall than foil triplets for high-value triplets, *t*(31) = 3.08, *p* = 0.004, Cohen’s *d =* 0.55, low-value triplets, *t*(31) = 2.25, *p* = 0.032, *d* = 0.39, and value-absent triplets, *t*(31) = 2.74, *p* = 0.01, *d* = 0.48.

To examine strength of evidence favoring the null hypothesis, we applied a Bayesian repeated-measures ANOVA these data using the JASP software project (Love et al., 2015), with default priors (Rouder et al., 2012). This analysis compares models that include versus do not include each factor and interaction, producing a Bayes Factor (BF) ratio that indicates the evidence in favor of the null model compared with evidence favoring a model that includes the factor or interaction in question. This analysis was used to produce a BF_01_ statistic for each main effect and the interaction. BF_01_ is an inverted Bayes Factor, with values greater than 1 indicating that the null model is favored, and with higher BF_01_ values indicating stronger evidence for a model that does not include the factor or interaction than one which does include the factor/interaction. In the base of both main effects and the interaction, evidence strongly favored the null model (target value main effect, BF_01_ = 17.4; foil value main effect, BF_01_ = 16.9; interaction, BF_01_ = 294.4), indicating strong evidence against the possibility that value meaningfully altered performance in the context of this experiment, either in terms of triplet or foil value.

## Experiment 1B

While we had chosen to pair the reward tone with the third item in every triplet with the intention of establishing a retroactive association to the triplet, it could be the case that the reward association enhances visual statistical learning for *subsequent* characters in the stream. In this case, any effect of reward would be washed out across randomized triplet orderings. We examined this possibility in Experiment 1B by instead providing a reward association with the first item in some triplets rather than the third. Other than this change, all other aspects of Experiment 1B were identical to Experiment 1A.

### Results

**Figure 3** displays mean accuracies in selecting target triplets over foil triplets for Experiment 1B. 3 × 3 repeated measures ANOVA, we found no significant interaction of target triplet value and foil triplet value, *F*(4,168) = 0.39, *p* = 0.82, η_p_^2^ = 0.009. No significant main effect of target value association was observed, *F*(2,84) = 0.57, *p* = 0.57, η_p_^2^ = 0.013, and no significant main effect of foil value association was observed, *F*(2,84) = 0.95, *p* = 0.39, η_p_^2^ = 0.022. Visual statistical learning, was again robust with participants correctly identifying more target triplets overall than foil triplets, *t*(42) = 4.97, *p* < 0.001, *d =* 0.76. We again applied a Bayesian repeated-measures ANOVA to assess strength of evidence favoring the null hypothesis, BF_01_. In the base of both main effects and the interaction, evidence continued to strongly favor the null model (target value main effect, BF_01_ = 16.8; foil value main effect, BF_01_ = 9.7; interaction, BF_01_ = 169.4.

## Experiment 2

Experiments 1A and 1B demonstrated no evidence that visual statistical learning processes are influenced by on-going reward signals paired consistently with constituent items, with no significant differences observed in identification accuracy according to value association. Experiment 2 was designed to explicitly introduce stimulus-reward learning prior to stimulus-stimulus associative learning, rather than including both on-going reward signals and stimulus-stimulus contingencies simultaneously. Participants were first required to learn the values of six specific symbols (half low-value, half high-value) at the start of the experiment. Instead of pairing a reward-tone with the symbols during the familiarization phase, participants in Experiment 2 were simply required to commit value associations of specific symbols to memory before beginning the familiarization phase.

In Experiment 2, stimulus-reward learning was induced by first showing participants all six symbols alongside their corresponding value (e.g., +1 or +9). This initial presentation occurred twice. Participants were then shown all six symbols sequentially, in a random order, and were required to press the “1” or the “9” key on the keyboard to indicate its value. Shuﬄed presentation of all six symbols comprised a single block, and before moving on to the familiarization phase participants were required to complete five consecutive blocks of the value-learning phase with 100% accuracy. At the beginning of this value-learning phase, participants were told that they would have to identify the value of a symbol at the conclusion of the experiment, and that they would be awarded the full value of that symbol if they are correct. Therefore, the value associations of these symbols was real, and participants’ ability to memorize their value would dictate whether or not they could win $1 or $9 at the conclusion of the study.

An attention-check task was also implemented in Experiment 2. However, instead of having participants press a spacebar whenever an item repeated, they were required to press the spacebar whenever an item “jiggled” from left to right during the familiarization phase. Additionally, because participants had already learned the value associations, the presentation of a value-associated tone was obviated. Instead, the three high-value (+9) and low-value (+1) symbols were placed as the first, second, or third item in six of the nine triplets, with position manipulated between groups. The remaining three triplets did not possess a symbol with a learned value association (i.e., none of the three neutral triplets’ shapes had been observed prior to the training phase).

During the test phase, the original triplet and the foil triplet were always matched by value. For example, a low-value original triplet was never paired with a high-value or a value-absent triplet. This logical restriction left us with a total of 54 test trials. Following the test phase, and congruent with what participants had been told in the initial value-learning phase, participants were required to recall the value of a symbol they had learned during the initial value-learning phase. One of the six value-associated symbols was presented on screen for people to explicit recall the value of. If a participant was correct in recalling the value of this symbol, they were rewarded with a corresponding dollar amount of either $1 or $9. Accuracy at this stage was 100% - all subjects responded correctly, verifying success of the reward-training regimen.

### Results and Discussion

**FIGURE 4:**
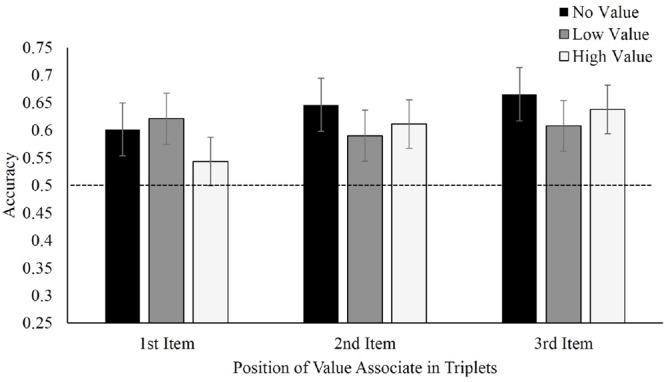
**Mean accuracy in identifying target triplets over foil triplets in Experiment 2, separated by position of value associate within the triplet**.

**Figure 4** displays mean accuracies in selecting target triplets over foil triplets for Experiment 2. With regard to where the value-associated symbols were placed, there was no difference in recall between value in the first, *F*(2,42) = 0.66, *p* = 0.52, η_p_^2^ = 0.03, second, *F*(2,34) = 0.45, *p* = 0.64, η_p_^2^ = 0.026, or third positions, *F*(2,40) = 0.48, *p* = 0.62, η_p_^2^ = 0.02. Nonetheless, participants continued to display visual statistical learning selecting learned triplets at above-chance levels, regardless of whether the learned symbol appeared in either the first, *t*(21) = 4.04, *p* = 0.001, *d =* 0.86, second, *t*(17) = 4.10, *p* = 0.001, *d =* 0.97 or third position *t*(20) = 7.02, *p* < 0.001, *d =* 1.53. Applying a Bayesian repeated-measures ANOVA for each value-associated placement again produced moderate evidence favoring the null model (first position, BF_01_ = 4.2; second position, BF_01_ = 4.8; and third position, BF_01_ = 5.0. Viewed as a mixed repeated-measures (value condition) and between-subjects (position) Bayesian ANOVA, the omnibus BF_01_ for the effect of target triplet value was 9.2.

## General Discussion

Based upon similar associative characteristics and impacts upon performance, we argued that stimulus-stimulus and stimulus-reward learning might interact. The shared neural basis of these two systems (Delgado et al., 2000; Aron, 2004; Wittmann et al., 2007; Lansink et al., 2009; Dickerson and Delgado, 2015) further bolstered our motivation to explore this possibility. Finally, the dependence of visual statistical learning on attention (Turk-Browne et al., 2005) in conjunction with the attention-modulating effects of reward (Engelmann et al., 2009; Hickey et al., 2010; Theeuwes and Belopolsky, 2012; Chelazzi et al., 2013; Sali et al., 2014; Pessoa, 2015), further suggest that reward learning may have the potential to enhance or disrupt statistical learning. However, results from the present study were unable to identify any influence of reward learning on visual statistical learning.

Despite reliable evidence that visual statistical learning successfully occurred throughout our experiments, we failed to find a reliable influence of reward. This may be most surprising in Experiment 1, where presentation of a sound stimulus probabilistically paired with either the first or third item provided additional subtle information about the presence of structure, as well as serving as a reward cue. Even in this case, where an additional cue to structure was present in value-associated triplets but absent from no-value triplets, participants performed comparably in identifying no-, low-, and high-value reward triplets. Thus, in an environment where reward learning and visual statistical learning are concurrently active, our results suggest that visual statistical learning is unaffected by the occurrence of rewarding events.

While visual statistical learning appears to be unaffected by concurrently active learning mechanisms, it also appears to be unaffected by previously learned reward. In Experiment 2, participants first learn to strongly associate values with symbols before engaging in the familiarization phase. Similar to our findings from the first experiment, visual statistical learning was generally evident across all conditions, but there appeared to be no clear effect of the value association upon learning, nor was there any effect of pre-exposure of some constituent shapes from the reward-learning phase of the experiment.

These results suggest that concurrently presented rewards and previously learned stimulus-reward associations have no impact on visual statistical learning. That is, whether stimulus-reward associations were introduced at the same time as stimulus-stimulus associations, or whether stimulus-reward associations were established before stimulus-stimulus associations were learned, participants’ ability to accurately identify familiar structured triplets of symbols remained unaffected. However, it is important to acknowledge the potential shortcomings of the present work.

It is possible that our attempt to create strong stimulus– reward contingencies was not powerful enough to influence visual statistical learning. In other words, it is feasible that one could have participants engage in a more rigorous reward-learning tasks before or during visual statistical learning. Some evidence suggests that the impact of reward on attention scales as rewards increase (Anderson et al., 2013). Given our understanding that visual statistical learning is dependent upon attention (Turk-Browne et al., 2005), one could argue that larger rewards may have produced a larger effect of reward learning by amplifying attention during high-reward experiences. However, potent and well-documented effects of reward on various cognitive processes have been demonstrated using similar reward values and reward delivery strategies (Cohen et al., 2007; Hickey et al., 2010; Chelazzi et al., 2013; Marx and Einhauser, 2015); thus, it seems unlikely that an interaction between reward learning and visual statistical learning was missed due to our choice of reward quantity, which is within the reasonable range of incentives provided in efforts that demonstrate clear effects of rewards.

In terms of reward’s effects on attention, one possible explanation for our null result is that attention actually has limited effect on visual statistical learning. Although earlier work suggested that statistical learning is “gated” by selective attention (Turk-Browne et al., 2005), recent work has challenged the robustness of this finding (Musz et al., 2015). Thus, it is possible that statistical learning is non-existent, context-dependent, or immune to variations in selective attention, or at least those variations likely evoked by the kinds of cues to reward used in our studies.

Regardless of the degree of attentional variation induced by the value manipulations used here, and of the role of attention in visual statistical learning, the well-established, strong role of value associations in driving variations in performance (possibly without necessitating a role of attention as a mediating variable) suggests potential for value associations to influence statistical learning. Two important considerations are the possibility of distinctions between primary and secondary reinforcers, and between transient- and state-based effects. Regarding the former, here we only tested secondary reinforcements (signals to monetary value). Primary rewards might be more powerful and effective at inducing changes to visual statistical learning. Regarding the latter, we have demonstrated cases in which reward-related stimuli selectively paired with constituent visual images does not impact visual statistical learning, but the random interleaving of low- and high-reward events leaves open the possibility that reward may influence statistical learning in a manner that depends upon cognitive or emotional state. An example of a well-known effect of emotional states on learning and memory are contextual effects of mood, in which better recall is experienced in a mood state congruent to encoding (Bower, 1981). Effects of reward on associative learning between stimuli could be state-based, require longer and more powerful periods of induction by repeated or otherwise more potent reward, and/or such effects may act over longer learning timescales.

The present works provides insight into the integrity of visual statistical learning, with evidence suggesting isolation from the effects of rewarding events. Additionally, while these two systems share similar neural correlates, the functional role of these neural structures in each type of learning may differ. However, we did not test the opposite relationship here – that statistical learning may impact reward learning, even if rewarding associations do not impact statistical learning. Statistical learning may precede reward learning and influence inferences about reward value (e.g., by supporting transitive inference of reward from one item to its statistical associates), or facilitate or impair reward learning in other ways. Further work is needed to explore these possibilities.

## Conclusion

Interjecting events that vary in reward significance appears to leave visual statistical learning unchanged. While this finding depends upon a lack of statistical significance, we saw no clear trend of any effect, implying limits to any such interference or facilitation. In environments that feature both statistical regularity amongst stimuli, as well as contingencies between those stimuli and rewarding events or history, our evidence suggests that statistical learning is unaffected and possibly independent of reward.

## Author Contributions

KF and TV conceived of, implemented, and collected data for the experiments. LR, KF, and TV analyzed and interpreted data. LR wrote the manuscript. LR, KF, and TV revised the manuscript.

## Conflict of Interest Statement

The authors declare that the research was conducted in the absence of any commercial or financial relationships that could be construed as a potential conflict of interest.
